# Selective state spectroscopy and multifractality in disordered Bose-Einstein condensates: a numerical study

**DOI:** 10.1038/s41598-018-21870-4

**Published:** 2018-02-26

**Authors:** Miklós Antal Werner, Eugene Demler, Alain Aspect, Gergely Zaránd

**Affiliations:** 1 Exotic Quantum Phases “Momentum” Research Group, Department of Theoretical Physics, Budapest University of Technology and Economics, 1111 Budapest, Budafoki út 8, Hungary; 2 Department of Physics, Harvard University, Cambridge, Massachusetts, 02138 USA; 3 Laboratoire Charles Fabry Institut d’Optique Graduate School – CNRS, Université Paris Sud, 2 avenue Augustin Fresnel, 91127 Palaiseau, France

## Abstract

We propose to apply a modified version of the excitation scheme introduced by Volchkov *et al*. on bosons experiencing hyperfine state dependent disorder to address the critical state at the mobility edge of the Anderson localization transition, and to observe its intriguing multifractal structure. An optimally designed, spatially focused external radio frequency pulse can be applied to generate transitions to eigenstates in a narrow energy window close to the mobility edge, where critical scaling and multifractality emerge. Alternatively, two-photon laser scanning microscopy is proposed to address individual localized states even close to the transition. The projected image of the cloud is shown to inherit multifractality and to display universal density correlations. Interactions – unavoidably present – are taken into account by solving the Gross-Pitaevskii equations, and their destructive effect on the spectral resolution and the multifractal spectrum is analyzed. Time of flight images of the excited states are predicted to show interference fringes in the localized phase, while they allow one to map equal energy surfaces deep in the metallic phase.

## Introduction

Anderson localization is one of the most fundamental quantum interference phenomena in disordered quantum systems. As first pointed out in the seminal work of Anderson^1^, all eigenstates of a particle on a disordered lattice localize in space in sufficiently strong disorder. Though this statement is independent of dimensionality, the existence of delocalized states and the nature of eigenstates at various energies still depend on the spatial dimension of the system considered, and may also be affected by the specific structure of the disorder^2–4^. While in *d* = 1 dimension all eigenstates are always localized for uncorrelated diagonal disorder, in dimensions *d* > 2 a critical disorder strength exists^5,6^. Below this critical disorder, mobility edges *E*_mob_ separating localized and delocalized states emerge. At the critical energies, *E* = *E*_mob_, a peculiar quantum phase transition takes place. Approaching *E*_mob_ through localized states, the typical spatial extension *ξ* of the wave functions (localization length) is found to diverge as a power law, $\xi (E)\sim |E-{E}_{{\rm{mob}}}{|}^{-\nu }$, with *ν* a universal exponent that depends only on dimensionality and the symmetry of the underlying Hamiltonian^7^. In case of *d* = 3 spatial dimensions and in the absence of external magnetic fields and spin-orbit interaction, studied here, the exponent *ν* = *ν*_orth_ = 1.58… has been determined with great accuracy by transfer matrix and finite-size-scaling methods, with the label’orth’ referring to orthogonal universality class^8,9^. At the critical energies, *E* = *E*_mob_, eigenstates of universal properties emerge; the absence of length scale, *ξ* → ∞ implies a self-similar character and entails a multifractal structure^10,11^ of the wave function^12–15^. In particular, at criticality, the probability distribution associated with the critical wave function *ψ*_**r**_ is predicted to scale with system size as

1 $$P(|{{\psi }}_{{\bf{r}}}{|}^{2}\sim {L}^{-\alpha })\propto {L}^{f(\alpha )-d},$$

with *f*(*α*) the universal multifractal spectrum of the critical wave function and *L* the system’s linear extension^16,17^. This multifractal structure has a deep origin: it is interpreted as a signature of the presence of infinitely many relevant operators at the Anderson transition^16^. Remarkably, multifractality is also suggested to emerge in the context of many-body localization: recent numerical studies of the Anderson problem on a random regular graph (RRG) reported the existence of the non-ergodic phase with eigenfunctions exhibiting multifractal structures with disorder dependent fractal dimensions^18–20^.

Though Anderson’s localization transition has been experimentally studied in a range of systems including doped semiconductors^21^, granular optical media^22–25^, disordered microwave waveguides^26^, elastic networks^27^, photonic lattices^28,29^, and cold atomic systems^30–34^, and it also emerges in driven quantum systems^35,36^, in spite of the considerable effort^37–39^, a convincing measurement of the critical state’s predicted universal multifractal spectrum remains missing.

In conventional solid state systems such as doped semiconductors, e.g., Scanning Tunneling Microscopy (STM) provides a way to gain information on the detailed structure of the critical wave function^38,39^. Indeed, in doped ferromagnetic semiconductors, emergent short distance power law signatures have been demonstrated close to criticality at the Fermi energy^38^, but the predicted anomalous exponent has not been observed and the computed multifractal spectra remained far from the theoretically predicted universal form. Rather, measured amplitude distributions exhibited close to lognormal distributions, characteristic of disordered metals. Moreover, in these systems, strong electron-electron interactions have a large impact on the metal-insulator transition, and Anderson’s non-interacting theory looses its validity^40,41^. STM signatures of multifractal properties have also been reported on iron-doped InAs surfaces in the context of quantum-Hall transition^39^, but the energy resolution was insufficient to observe the mulifractal spectrum of the critical quantum-Hall state^42^. Indications of multicriticality have also been reported in ultrasound experiments^37^ but, the universal multifractal spectrum of the critical state has not been observed in these experiments, either^37,43^.

Experiments with ultracold atoms provide powerful and versatile means to realize and study the Anderson transition. In these experiments laser speckles^32,33^, quasiperiodic optical lattices^31^, or holographic methods^44–46^ can be used to produce and design disorder, and the interaction of the atoms can be controlled by changing the depth of an applied optical lattice, the strength of the confinement potential or by using Feschbach resonances. The localization transition has been observed in one- and three-dimensional systems^30–34^, and weak localization and coherent back scattering in two dimensions has also been investigated experimentally^47,48^. Cold atomic experiments should also allow to observe novel dynamical features, such as the the emergence of the so-called coherent forward scattering (CFS) peak in the Anderson localized phase^49,50^. None of the existing experiments could, however, detect the critical state so far. In fact, addressing the critical state turns out to be a very delicate task: importantly, the multifractal behavior is the property of a *single* eigenstate and, as we shall see, admixture of just a few eigenstates by interactions or inelastic processes even in a narrow energy window makes it hard to observe.

**Figure 1:**
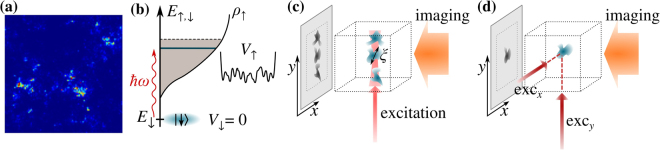
(**a**) Projected image of a critical multifractal eigenstate of size (*L*/*a*)^3^ = 100^3^, exhibiting clustering. (**b**–**d**) Schematics of the proposed experiment: (**b**) The condensate is prepared in the hyperfine state |↓〉 where no disorder is present. The external field excites the atoms to the hyperfine state |↑〉, interacting with the disorder potential *V*_↑_. The energy *E*_↑_ of the final state is controlled by the frequency *ω*. (**c**) Excited atoms in the |↑〉 state are imaged by a horizontal laser beam. The overlap between different eigenstates can be reduced by using a narrow excitation beams of waist ${w}_{0}\lesssim \xi$, where *ξ* is the typical size of a localized state. (**d**) Even a single localized state can be excited and imaged by crossing two laser beams.

Here we propose to image the critical state and to detect its multifractal properties in the particular cold atomic setups, sketched in Fig. 1. Following the method proposed and demonstrated by Volchkov *et al*.^51^, we suggest to use bosons with two hyperfine components, *σ* = ↑ and ↓, with only the component ↑ being subject to a random potential *V*_↑_. Placing all bosons initially in the weakly interacting (and homogeneous) state ↓ and exciting from this spin ↓ condensate spin ↑ states with the external frequency *ω*, the energy *E*_↑_ = *E*_↓_ + *ħω* of the final state can be selected and tuned through the mobility edge of the spin ↑ component either by changing *ω* or by varying the disorder amplitude. As we demonstrate through detailed large scale simulations, it is possible to single out only a few final states in a very narrow energy window by (1) carefully optimizing the shape of the excitation pulse, (2) switching off interactions in the final state, and (3) by using carefully designed excitation geometries.

In particular, we propose to use two different arrangements. In the first geometry, we propose to use a thin vertical laser beam with two frequencies to produce two-photon Raman transitions to localized states in a narrow spatial range and at a well-defined energy such that that the excited wave functions do not overlap. Projection imaging along the horizontal direction (see Fig. 1c) yields images of non-overlapping localized wave functions close to the mobility edge *E*_mob_. Alternatively, instead of one thin vertical beam with two frequencies, one could use *crossed* laser beams to address *individual localized* states via two-photon Raman transitions in just the small crossing region (see Fig. 1d). This method, developed in biological microscopy and known as ‘Two Photon Laser Scanning Microscopy’ (TPLSM)^52^, is similar in spirit to the one proposed by Kollath *et al*.^53^, but here no probe atoms are needed. Rather, transitions are generated from a Bose condensate of atoms, and the local character of the transitions is ensured through crossing the two laser beams.

As we show below through detailed simulations, multifractal properties of the critical state are *inherited* by the projected pictures of localized states with sufficiently large localization lengths. The projection of the squared amplitude of wave function, i.e., the density exhibits a non-trivial multifractal spectrum which we determine through large scale simulations, and the projected density-density correlation functions are predicted and demonstrated to display universal scaling collapse, thereby providing evidence for multifractal behavior.

## Results

In the absence of radiation, we can describe the two-component Bose mixture in the optical lattice by the Hamiltonian,

2 $$H=-J\sum _{{\bf{r}},{\bf{r}}{\boldsymbol{^{\prime} }},\sigma }^{\prime} {a}_{{\bf{r}}\sigma }^{\dagger }{a}_{{\bf{r}}{\boldsymbol{^{\prime} }}\sigma }+\sum _{{\bf{r}},\sigma }{\varepsilon }_{{\bf{r}}\sigma }{a}_{{\bf{r}}\sigma }^{\dagger }{a}_{{\bf{r}}\sigma }+\sum _{{\bf{r}},\sigma ,\sigma ^{\prime} }\frac{1}{2}{U}_{\sigma \sigma ^{\prime} }{a}_{{\bf{r}}\sigma }^{\dagger }{a}_{{\bf{r}}\sigma ^{\prime} }^{\dagger }{a}_{{\bf{r}}\sigma ^{\prime} }{a}_{{\bf{r}}\sigma },$$

with the prime indicating summation over neighboring sites only. The on-site energies *ε*_**r***σ*_ incorporate the chemical potential, and *ε*_**r**↑_ also contains the random component *V*_↑_(**r**), responsible for Anderson localization of the ↑ bosons. In practice, the random potential *V*_↑_(**r**) is realized by fine-grained speckle potentials^54^ or holographic disorder^46^. Here, for simplicity, we shall replace it by a uniform and independent distribution of *ε*_**r**↑_∈[−*W*/2, *W*/2] on each lattice site.

A possibility to generate hyperfine ↑ ⇒ ↓ transitions in a sufficiently narrow spatial region is to use two-photon processes^52^. Here we consider a stimulated Raman process^51^, which generates an effective RF field with a frequency corresponding to the beating frequency of the two lasers, and can be described in the rotating frame approximation by the coupling term

3 $${H}_{{\rm{\Omega }}}=\sum _{{\bf{r}}}\hslash {{\rm{\Omega }}}_{{\bf{r}}}(t)\,({a}_{{\bf{r}}\uparrow }^{\dagger }{a}_{{\bf{r}}\downarrow }+h\mathrm{.}c\mathrm{.),}$$

with Ω_**r**_(*t*) the Rabi frequency, and the RF frequency appearing simply as an energy shift *ε*_↑, ↓_ → *ε*_↑, ↓_ ± *ħω*/2. For Ω_**r**_(*t*) we assume a Gaussian profile, ${{\rm{\Omega }}}_{{\rm{r}}}\sim {{\rm{\Omega }}}_{0}(t){e}^{-2({x}^{2}+{y}^{2})/{{w}_{0}}^{2}}$ with a narrow waist *w*_0_ in the range of a few lattice constants, ${w}_{0}\sim 2\,\mu m$ The shape of the pulse Ω_0_(*t*) must be determined carefully to generate transitions between the two hyperfine components in an energy window as narrow as possible. Since the coupling happens between a discrete state and a quasi-continuum, the width of the final energy window follows from Fermi’s Golden rule^55^, and can be adjusted by the duration and shape of the pulse Ω(*t*). However, interactions in the final state turn out to be particularly destructive: they generate unwanted transitions and broaden the spectrum of the final state. Therefore, in the following, we shall assume that interactions in the final state have been switched off by applying an appropriate external magnetic field, *U*_↑↑=0_. We assume further that the two hyperfine states are decoupled for *t* < 0, Ω_0_(*t* < 0) = 0, and the bosons form a condensate in the state ↓, described by a collective wave function *a*_**r**,*σ*_ → *ψ*_**r***σ*_ at time *t* = 0. We describe the time dependence of this collective wave function in terms of the Gross-Pitaevskii equation (see Methods).

### Numerical simulations

We determined the time dependence of the collective wave function by solving Eq. (10) numerically. To maintain numerical accuracy, we suppressed fast phase oscillations of the wave function by means of a self-consistently determined global gauge transformation (see Eq. (12) in Methods). This allowed us to perform Gross-Pitaevskii simulations on systems as large as 40 × 40 × 40, where length is measured in units of the lattice constant *a*. Since the system size is not larger than the Rayleigh length $\pi {w}_{0}^{2}/\lambda$ of the laser beam, we neglected the spatial spreading of the waist, and assumed a simple cylindrical beam. The RF field Ω_**r**_(*t*) was turned on smoothly, kept constant and then turned off with an optimized pulse of length *T* = 400 *ħ*/*J* and turn on time *τ* = 60 *ħ*/*J*. This resulted in an energy window Δ*ε* of the final states encompassing a limited number of states (see the discussion below).

In small disordered systems, disorder averaging is necessary to eliminate sample to sample fluctuations. Averaging over a huge ensemble of disorder realizations is experimentally demanding. Disorder averaging can, however, also be replaced in part by averaging over the RF frequency *ω* in a small window, since the localization length *ξ* (E) displays only a weak energy dependence near the band center^5^.

**Figure 2:**
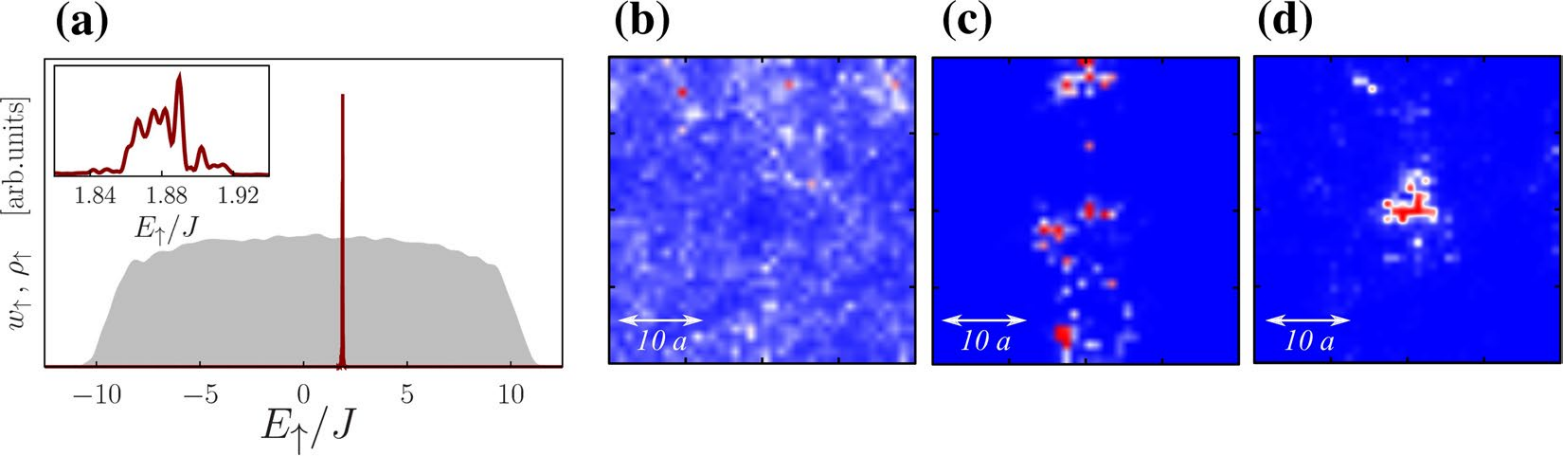
(**a**) Spectral weight (red line) *w*_↑_(*E*_↑_) of the ↑ bosons as a function of the final state’s energy *E*_↑_ after the excitation pulse for a lattice of (*L*/*a*)^3^ = 40^3^ sites, *W* = 17*J* and excitation beam waist *w*_0_ = 4*a*. We simulated *N* = 3200 atoms with typical interaction strengths *U*_↑↓_ = *U*_↓↓_ = 2*J*, excitation frequency *ω* ≈ 8*J*/*ħ*. The grey area indicates the smoothed density of states of the ↑ bosons in the self-consistent disorder potential that combines the static external disorder and the repulsion of the ↓ background. Inset: same spectral peak on a smaller scale. (**b**,**c**) Projected images of a metallic (*W* = 10*J*) and a localized (*W* = 28*J*) final state. The excitation beam has vertical direction in both cases. (**d**) Projected image of a single, slightly localized state (*W* = 17*J*), excited by two crossed laser beams. All figures show images of size 40*a* × 40*a*.

The spectral and spatial structure of the state reached after the excitation pulse is shown in Fig. 2 for some typical parameters. As shown in panel (a), states are excited in a very narrow energy window. To determine the spectral weights *w*_↑_ (*E*) and the density of states, displayed in panel (a), we employed the so-called Chebyshev polynomial expansion (see Methods), which made possible to obtain high accuracy results and thus circumvent the impossible task of performing a complete diagonalization of the final Hamiltonian. Although states in a very narrow window of width ${\rm{\Delta }}\varepsilon \sim 0.05\,J$ could be excited by means of optimizing the pulse shape and using a small concentration of atoms, still, around 100 eigenstates can be found in this tiny interval even in a relatively small 40 × 40 × 40 lattice, containing 64,000 lattice sites. Mixing 100 eigenstates would make the multifractal properties completely invisible. Indeed, on the metallic side, states are uniformly excited over the whole sample (see Fig. 2b), and exciting and imaging of individual states does not seem to be possible.

Fortunately, as shown in Fig. 2c, this problem can be avoided on the localized side of the transition by applying narrow laser beams of waist *w*_0_ < *ξ*, and thereby reducing the number of excited eigenstates by a factor $\sim {(\xi /L)}^{2}$, where *L* denotes the linear size of the system. These localized states are excited along the waist of the laser beam, but they do not spatially overlap under the condition

4 $${\rm{\Delta }}\varepsilon  < \frac{1}{{\rho }_{\varepsilon }{\xi }^{3}},$$

with *ρ*_*ε*_ standing for the the average density of states per unit volume of spin ↑ bosons. To derive this inequality, we notice that a narrow laser beam excites states only in a column of volume *ξ*^2^*L*. Consequently the total number of excited states is $N\sim {\xi }^{2}L\,{\rho }_{\varepsilon }{\rm{\Delta }}\varepsilon$, and the average spatial distance between these states is $d\sim L/N$. The overlap of the distinct excited states in the image is thus small if *d* > *ξ*, which leads us to the condition Eq. (4). This condition — meaning that the energy resolution be better than the level spacing in a localization volume — can be satisfied even relatively close to the transition, but fails for typical parameters very close to the transition and in the metallic phase. The same condition is obtained for the TPLSM protocol (Fig. 1d). We thus conclude that imaging individual states is possible on the localized side of the transition with the suggested protocols as long as Eq. (4) remains satisfied.

### Multifractal properties

The statistical properties of the critical state reflect the structure of the localization phase transition. Similar to classical critical correlations, close to the transition, the disorder-averaged correlation functions of the *q*-th moments of the density display critical scaling^16^,

5 $${C}_{\uparrow }^{(q)}({\bf{r}})\equiv \overline{|{\psi }_{{\bf{r}}\uparrow }{|}^{2q}|{\psi }_{0\uparrow }{|}^{2q}}\propto |{\bf{r}}{|}^{-{y}_{q}}\,{Y}^{(q)}({\bf{r}}/\xi \mathrm{).}$$

At criticality, the localization length *ξ* diverges, yielding power law correlations with *q* dependent exponents, *y*_*q*_. Close to criticality these power law correlations are cut off beyond the localization length (correlation length), as described by the universal cut-off function *Y*^(*q*)^(*r*/*ξ*). The exponent *y*_*q*_ here turns out to be intimately related to the Legendre transform *τ*_*q*_ of the multifractal spectrum *f*(*α*)

6 $${y}_{q}=2{\tau }_{q}-{\tau }_{2q}+d,$$

with $q\equiv \frac{{\rm{d}}f(\alpha )}{{\rm{d}}\alpha }$, τ_*q*_ = *αq* − *f*(*α*), and *d* = 3^16^.

The previous discussion we considered three dimensional wave functions and densities, which are quite difficult to determine experimentally^56^. Fortunately however, as we now show, it is also possible to observe the fingerprints of multifractality in the experimentally measured *projected* densities of the gas,

7 $${\hat{\rho }}_{x\sigma }\equiv \int dz|{\psi }_{{\bf{r}}\sigma }{|}^{2}$$

with **x** = (*x*,*y*) referring here to coordinates in the plane of projection. While the rare regions of small amplitudes (large *α*) appear to be washed out by the projection and multifractality is destroyed there, multifractal scaling and power law correlations are found to survive the projection in the large amplitude (small *α*) regions and are inherited by the projected wave function. For *q* = 1, in particular, it is easy to prove (see Supplemental Material) that the projected densities display power law correlations with an anomalous exponent

8 $${\hat{y}}_{q=1}={y}_{1}-1\approx 0.8,$$

providing a clear analytical evidence for the survival of multifractality. This relation is indeed verified by our numerics (see Supplemental Material), which also confirm that the projected densities ${\hat{\rho }}_{{\bf{x}}\uparrow }$ display multifractal scaling according to Eqs (1) and (5) with, however, a modified multifractal spectrum, $\hat{f}(\alpha )$, and modified exponents ${\hat{y}}_{q}$ and correlation functions

9 $${\hat{C}}^{(q)}({\bf{x}})\equiv \overline{{\hat{\rho }}_{{\bf{x}}\uparrow }^{q}{\hat{\rho }}_{0\uparrow }^{q}}\propto |{\bf{x}}{|}^{-{\hat{y}}_{q}}{\hat{Y}}^{(q)}({\bf{x}}/\xi \mathrm{).}$$

**Figure 3:**
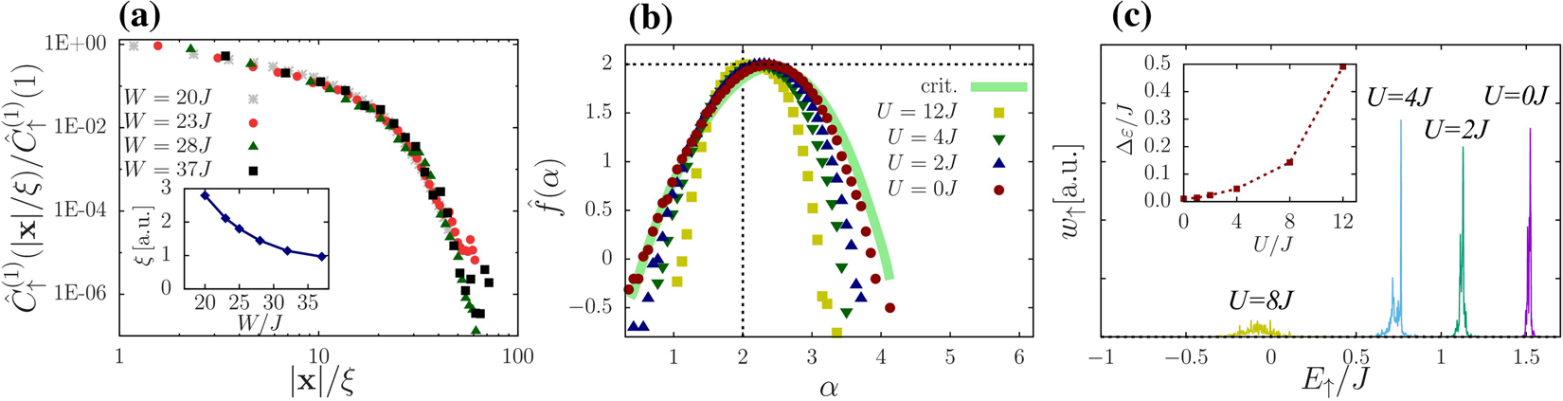
(**a**) Rescaled correlation function of the projected densities ${\hat{\rho }}_{{\bf{x}}\uparrow }$ for different disorder strengths but fixed excitation frequency *ω* ≈ 8*J*/*ħ* in the localized regime. Correlations were measured along the *y* direction in Fig. 1. Other parameters were set as in Fig. 2. Values of *ξ*(*W*) were determined by collapsing the curves. Inset: divergence of *ξ* at *W* → *W*_*c*_ ≈ 16.5*J*. (**b**) Multifractal spectrum extracted from the probability distribution of the projected density ${\hat{\rho }}_{\uparrow }$. The measured spectrum of non-interacting bosons excited by a short pulse (red circles) follows closely the projected spectrum of the critical state (thick green line). The location of the maximum, *α*_m*ax*_ ≈ 2.3, characterizes the anomalous scaling of the typical projected density, ${\hat{\rho }}_{{\bf{x}}\uparrow }^{{\rm{typ}}}\sim {L}^{-{\alpha }_{{\rm{\max }}}}$. The multifractal spectrum gets distorted upon increasing interaction strength. (**c**) Spectrum of the final state for a fixed disorder configuration as a function of *U*/*J*. The sharp spectral peaks are shifted and broadened with increasing interaction, leading to eigenstate overlaps in the projected images and resulting in the degradation of the measured multifractal spectrum. Inset: average spectral peak width (full width at half maximum) as a function of *U* for *N* = 3200.

Figure 3a displays the correlation functions of the horizontally projected densities for different disorder strengths after the application of the excitation pulse. The agreement with (9) is demonstrated by a scaling collapse of the measured correlation functions: rescaling the separation |**x**| by a disorder dependent length scale, all normalized correlation functions fall onto a *single* universal curve. The emerging length scale can be identified as the localization length, and it indeed appears to diverge as we approach the localization transition at *W*_*C*_ (see inset of Fig. 3a). While the universal scaling collapse of the projected correlations is quite convincing, it appears to be hard to extract the precise value of the exponents ${\hat{y}}_{q}$ from ${\hat{C}}^{(q)}({\bf{x}})$.

Instead of studying correlation functions, we can also extract the multifractal spectrum directly from the probability distribution of the projected wave functions. We extracted $\hat{f}(\alpha )$ simply by measuring the distribution of $\mathrm{ln}({\hat{\rho }}_{{\bf{x}}\uparrow })$ after a pulse targeting the critical state. The final results are shown in Fig. 3b. In the absence of interactions, the spectrum of the state reached after the Rabi pulse follows very closely the spectrum of the true projected critical state itself. The maximum of $\hat{f}(\alpha )$ characterizes the scaling of the typical wave function amplitudes at criticality, and the fact that *α*_max_ > 2 is a clear evidence of the fractal nature of the projected wave function. Notice that for $\alpha \lesssim {\alpha }_{{\rm{\max }}}^{3D}-1\approx 3$ we do not expect a true multifractal spectrum: large values of *α* correspond to tiny wave function amplitudes which would be mixed with larger, typical wave function amplitudes upon projection, leading to the destruction of multifractality in the large *α* region. Indeed, a finite size analysis seems to confirm that the $\hat{f}(\alpha )$ function is well defined only below a critical value of *α* ≈ 2.5. We should also emphasize that the multifractal spectrum $\hat{f}(\alpha )$ is clearly different from the surface multifractal spectrum of the critical Anderson state^43^ (see Supplementary Material), which is supposed to be measured in an STM experiment^38^ or in the ultrasound setup of ref.^37^.

Having the spectra $\hat{f}(\alpha )$ (and *f*(*α*)) at hand, we can also perform a Legendre transformation to determine the critical exponents ${\hat{\tau }}_{q}$ and ${\hat{y}}_{q}$ (*τ*_*q*_ and *y*_*q*_), and compare them to the exponents extracted directly from the correlation functions (see Supplemental Material). In particular, for *q* = 1 we obtain *y*_1_ ≈ 1.8 and ${\hat{y}}_{1}\approx 0.8$, in good agreement with the exponents extracted from the scaling collapse of the density correlation functions, thereby verifying the exact relation (8).

As shown in Fig. 3b, in the non-interacting case one can excite the critical state with high precision, and observe the projected multifractal spectrum. Increasing the interaction leads, however, to deviations and ultimately destroys the spectrum. Notice that the relevant, small *α* region of the spectrum (describing high density fluctuations) remains relatively robust up to interactions of the order of *U* = 4*J*, but the rare event region, $\alpha \lesssim 2.5$ is quickly degraded.

The observed degradation of $\hat{f}(\alpha )$ is due to the interaction of the two hyperfine species: atoms removed from the ↓ condensate leave behind a hole of irregular shape in the atomic density, *δρ*_↓_(**r**,*t*). The missing atoms create then an effective time dependent random potential *U*_↑↓_*δρ*_↓_(**r**,*t*). Though this potential is quite shallow for intermediate interaction (see Supplemental Material), it still broadens the spectrum of the final state, as shown in Fig. 3c. Mixing of too many states leads to an overlap of the final wave functions, and amounts to the degradation observed.

We need to mention two further experimental aspects that may reduce the visibility of the multifractality of the excited condensate. High resolution imaging techniques usually correspond to strong measurements on the position of individual atoms^56–58^. One typically cannot therefore access ${\hat{\rho }}_{{\bf{x}}\uparrow }$ through a single shot measurement, rather, one has to prepare the same wave function multiple times and average the measured densities to obtain ${\hat{\rho }}_{{\bf{x}}\uparrow }$. High density regions, in particular, can be measured accurately, while low density regions are less visible. Fortunately, the multifractal spectrum depends only *logarithmically* on these statistical fluctuations. Therefore, as shown in the Supplemental Material, already hundreds of measurements on the same wave function can be sufficient to extract ${\hat{\rho }}_{{\bf{x}}\uparrow }$ and reveal $\hat{f}(\alpha )$. Particle number fluctuations may also lead to trouble: such fluctuations influence the background potential and lead to a slight detuning of the final state in each measurement. As shown in the Supplemental Material, below few percent particle number fluctuations are, however, not detrimental, and do not destroy the multifractal spectrum. We remark that post-selection is a natural possibility to reduce the error induced by particle number fluctuations.

### Time of flight images

**Figure 4:**
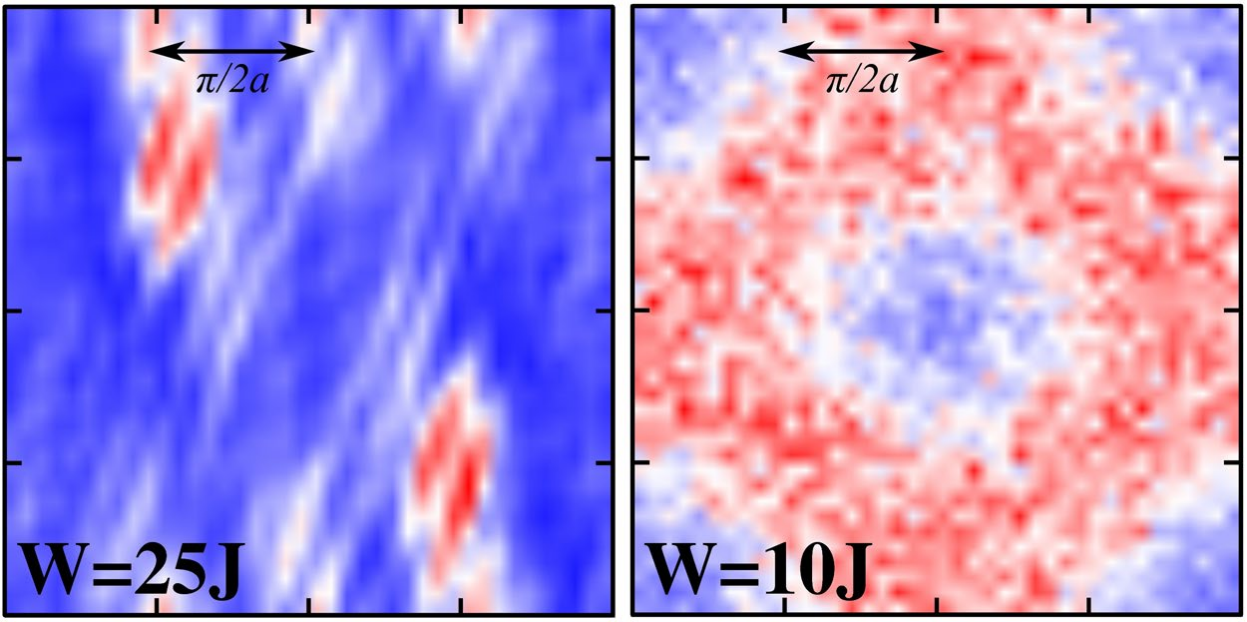
Time of flight image of excited atoms in the localized (left) and in the delocalized phase (right). The approximate self-consistent energies of the shown localized and delocalized states are *E*_↑_ = 1.9 *J* and *E*_↑_ = 0.6 *J* respectively. In the localized phase interference fringes emerge, while in the delocalized phase a “Fermi surface” structure tracing smeared equal energy surfaces in momentum space appears.

While time of flight images do not show any particular features at criticality, they do exhibit peculiar structures on both sides of the localization transition. For strongly localized final states, in particular, excited atoms happen to localize at only a few resonant localized states. Releasing the atoms from the trap then leads to interference fringes created by atoms released from these localized states, similar to the fringes observed in the case of split condensates^59^ (see Fig. 4). In the delocalized phase, on the other hand, weak disorder leads to the mixing of states on a relatively narrow energy shell, and, correspondingly, “Fermi surface”-like features emerge in the time of flight image, tracing approximately the equienergetic surfaces in the former Brillouin zone.

## Discussion

In this work, we carried out detailed large scale Gross-Pitaevskii simulations to investigate selective final state spectroscopy in an interacting disordered two-component Bose condensate. We proposed particular experimental protocols making possible to image and observe single localized states as well as the so far elusive Anderson critical state using weakly interacting ultracold bosons with two hyperfine states. To reveal the critical correlations and amplitude fluctuations, one needs to minimize the effect of interactions, and excite atoms into a few eigenstates in an extremely narrow energy window, otherwise the fragile multifractal structure becomes completely washed out. Our detailed simulations verify the feasibility of the proposed experiment. Three important ingredients were however needed to reach the required energy resolution: (1) interactions in the final hyperfine state were suppressed, (2) the shape of the excitation pulse has been optimized, and (3) excitation beams narrower than the localization length have been combined with a horizontal imaging to reduce the number of excited eigenstates on the localized side of the transition. Combining these three ingredients, one can reach energy resolutions sufficient to observe even single localized states and to detect critical multifractal correlations as well as multifractal amplitude fluctuations, even after projecting the image of the excited cloud.

We have verified that the projected image inherits the multifractal structure of the bulk. In particular, we have determined the *multifractal spectrum* of the image in the case of orthogonal symmetry, and predicted and have demonstrated by our simulations a universal power low scaling for the projected density-density correlations as a clear signature of multifractality. We believe that our protocol gives a way to go beyond earlier experimental attempts^37–39^, and observe the theoretically predicted (projected) multifractal exponents.

Time of flight images of the excited states have also been shown to display interesting features in both phases: strong localization-induced interference fringes appear in the localized phase, while momentum space equal energy surfaces can be imaged on the delocalized side of the transition.

Cold atomic systems and the method proposed here thus provide a unique framework to reach the critical state of Anderson localization and to study its multifractal properties, observed solely numerically so far. The proposed method is, of course, not restricted to Anderson’s localization transition. It can also be used to shed light on the critical state of disordered quantum Hall systems^60^, disordered systems with spin-orbit coupling^61,62^, or even more exotic topological phase transitions^63,64^. The proposed crossed beam two-photon laser scanning imaging setup would allow one to create and study local excitations of exotic and interacting quantum states.

## Methods

### Gross-Pitaevskii simulation

The time evolution of the collective wave function is described by the mean field Gross-Pitaevskii equation,

10 $$i{\partial }_{t}{\psi }_{{\bf{r}}\sigma }=\frac{\partial H(\{{\psi }_{{\bf{r}}\sigma }^{\ast },{\psi }_{{\bf{r}}\sigma }\})}{\partial {\psi }_{{\bf{r}}\sigma }^{\ast }}.$$

Substituting the Hamiltonian (Eq. (2)) in the above expression we arrive at the equations

11 $$\begin{array}{rcl}i{\partial }_{t}{\psi }_{{\bf{r}},\uparrow } & = & -J\sum _{{\bf{r}}^{\prime} }^{\prime} {\psi }_{{\bf{r}}^{\prime} ,\uparrow }+({V}_{\uparrow }(r)-\omega /2){\psi }_{{\bf{r}},\uparrow }+{{\rm{\Omega }}}_{{\bf{r}}}(t){\psi }_{{\bf{r}},\downarrow }+{U}_{\uparrow \downarrow }{|{\psi }_{{\bf{r}},\downarrow }|}^{2}{\psi }_{{\bf{r}},\uparrow }\\  & \equiv  & \sum _{{\bf{r}}^{\prime} }{\hat{H}}_{\uparrow }{(\psi )}_{{\bf{rr}}^{\prime} }{\psi }_{{\bf{r}}^{\prime} ,\uparrow }+{{\rm{\Omega }}}_{{\bf{r}}}(t){\psi }_{{\bf{r}},\downarrow },\\ i{\partial }_{t}{\psi }_{{\bf{r}},\downarrow } & = & -J\sum _{{\bf{r}}^{\prime} }^{\prime} {\psi }_{{\bf{r}}^{\prime} ,\downarrow }+\frac{\omega }{2}{\psi }_{{\bf{r}},\downarrow }+{{\rm{\Omega }}}_{{\bf{r}}}(t){\psi }_{{\bf{r}},\uparrow }+({U}_{\uparrow \downarrow }{|{\psi }_{{\bf{r}},\uparrow }|}^{2}+{U}_{\downarrow \downarrow }{|{\psi }_{{\bf{r}},\downarrow }|}^{2}){\psi }_{{\bf{r}},\downarrow }\\  & \equiv  & \sum _{{\bf{r}}^{\prime} }{\hat{H}}_{\downarrow }{(\psi )}_{{\bf{rr}}^{\prime} }{\psi }_{{\bf{r}}^{\prime} ,\downarrow }+{{\rm{\Omega }}}_{{\bf{r}}}(t){\psi }_{{\bf{r}},\uparrow }.\end{array}$$

with the primes indicating summations over neighboring lattice sites. In our simulations, the time-derivative of the wave function is dominated by a rapidly changing overall phase that can be easily removed by the gauge transformation

12 $${{\rm{\Phi }}}_{{\bf{r}}\sigma }(t)\equiv {e}^{i{\int }_{0}^{t}{E}_{sc}({\rm{\Psi }})dt^{\prime} }{\psi }_{{\bf{r}}\sigma }(t)\,,\quad {\rm{with}}\quad {E}_{sc}({\rm{\Psi }})=\frac{\sum _{\sigma ,{\bf{r}}}{\psi }_{{\bf{r}}\sigma }^{\ast }i{\partial }_{t}{\psi }_{{\bf{r}}\sigma }}{\sum _{\sigma ,{\bf{r}}}{\psi }_{{\bf{r}}\sigma }^{\ast }{\psi }_{{\bf{r}}\sigma }}\mathrm{.}$$

The equation of motion for Φ_**r***σ*_(*t*) is then solved numerically by using a standard 4th-order Runge-Kutta scheme.

### Chebyshev method for spectral properties

To get spectral information about the final state Φ_**r**↑_(*t*_end_) the kernel polynomial method is used^65^. At the end of the excitation process the external RF field is turned off, Ω_**r**_(*t*_end_) = 0, and the mean-field Hamiltonian has the diagonal form

13 $$\hat{H}(\psi )=(\begin{array}{ll}{\hat{H}}_{\uparrow }(\psi ) & 0\\ 0 & {\hat{H}}_{\downarrow }(\psi )\end{array}).$$

The single particle density of states for the ↑ bosons is approximated by the series

14 $${\rho }_{\uparrow }({E}_{\uparrow })=\sum _{\alpha }\delta ({E}_{\uparrow }-{E}_{\alpha })\approx \sum _{n\mathrm{=0}}^{{n}_{{\rm{\max }}}}{g}_{n}^{({n}_{{\rm{\max }}})}{\mu }_{n}{T}_{n}(2\frac{{E}_{\uparrow }-{\rm{BC}}}{{\rm{BW}}}),$$

where *E*_*α*_ denotes the energy of the eigenstate $|\alpha \rangle$ of ${\hat{H}}_{\uparrow }(\psi )$, *T*_*n*_(*x*) stands for the *n*’th Chebyshev polynomial and *μ*_*n*_ denote the Chebyshev expansion coefficients, which can be efficiently determined using the recursion relation for the Chebyshev polynomials^65^. The spectrum is transformed to the [−1,1] interval by shifting energies to the band center, B*C* and normalizing them by the bandwidth B*W* of the mean-field Hamiltonian. The factors ${g}_{n}^{({n}_{{\rm{\max }}})}$ are the expansion coefficients of the so called kernel function^65^ that regularizes the finite order approximation of the *δ*-functions. In our calculations we used the Lorentz kernel ${g}_{n}^{({n}_{{\rm{\max }}})}=\,\sinh (\lambda \mathrm{(1}-n/{n}_{{\rm{\max }}}))/\,\sinh (\lambda )$ that guarantees a positive definite density of states^65^. The parameter of the kernel was set to *λ* = 0.1.

A similar calculation is performed to approximate the spectral distribution of the final state of the Gross-Pitaevskii simulation yielding

15 $${w}_{\uparrow }({E}_{\uparrow })=\sum _{\alpha }|\langle \alpha |{{\rm{\Phi }}}_{\uparrow }\rangle {|}^{2}\delta ({E}_{\uparrow }-{E}_{\alpha })\approx \sum _{n\mathrm{=0}}^{{n}_{{\rm{\max }}}}{g}_{n}^{({n}_{{\rm{\max }}})}{\nu }_{n}{T}_{n}(2\frac{{{\rm{E}}}_{\uparrow }-{\rm{BC}}}{{\rm{BW}}}),$$

with $|{{\rm{\Phi }}}_{\uparrow }\rangle$ denoting the wave function of the ↑ bosons in the final state. The cutoff was set to values as large as *n*_max_ = 30,000 to resolve the sharp spectral peak of the final state.

### Data availability

The data sets generated during and/or analyzed during the current study are available from the corresponding author upon reasonable request.

## Electronic supplementary material

Supplemental Material
