# Supplementary material for: Selective state spectroscopy and multifractality in disordered Bose-Einstein condensates: a numerical study
Source: Sci Rep. 2018 Feb 26;8:3641. doi: 10.1038/s41598-018-21870-4 (PMC5832171; doi:10.1038/s41598-018-21870-4)
Supplement: Supplementary file 1 — Supplemental Material [file 41598_2018_21870_MOESM1_ESM.pdf]

Supplemental Material to "Selective state spectroscopy and multifractality in disordered Bose-Einstein condensates: a numerical study"

Miklós Antal Werner[1,*], Eugene Demler[2], Alain Aspect[3], and Gergely Zaránd[1]

[1]Exotic Quantum Phases "Momentum" Research Group, Department of Theoretical Physics, Budapest University of Technology and Economics, 1111 Budapest, Budafoki út 8, Hungary
[2]Department of Physics, Harvard University, Cambridge, Massachusetts 02138, USA
[3]Laboratoire Charles Fabry Institut d'Optique Graduate School – CNRS – Université Paris Sud, 2 avenue Augustin Fresnel, 91127 Palaiseau, France
[*]werner@phy.bme.hu

January 31, 2018

1 Numerical analysis of projected critical eigenstates

As a reference, we first compared the properties of the wave functions obtained by our time dependent Gross-Pitaevskii simulation with those of the critical eigenstate of the 3D Anderson model,

$$\hat{H}_{\rm AM} = -J \sum_{\mathbf{r},\mathbf{r}'}{}' a^\dagger_{\mathbf{r}} a_{\mathbf{r}'} + \sum_{\mathbf{r}} \varepsilon_{\mathbf{r}} a^\dagger_{\mathbf{r}} a_{\mathbf{r}} \;. \tag{S.1}$$

Here the prime indicates that the first sum runs over nearest neighbor sites on a 3D cubic lattice only. The operators $a^\dagger_{\mathbf{r}}$ ($a_{\mathbf{r}}$) create (annihilate) a particles at site $\mathbf{r}$, and the on-site energies $\varepsilon_{\mathbf{r}}$ are uniformly distributed in the interval $[-W/2, W/2]$. We used the JADAMILU library [1] to extract the exact critical eigenstates of the Anderson model up to linear system sizes $L = 120$ (i.e. $\sim 1.7\ 10^6$ sites), where length is measured in units of the lattice constant a, and analyzed their multifractal properties.

Having obtained the exact critical eigenstates of (S.1), we determined their projected densities $\hat{\rho}_{\mathbf{x}}$. Assuming that $\hat{\rho}_{\mathbf{x}}$ displays $\hat{d} = 2$ dimensional multifractality, we expect its probability density to scale as

$$P(\hat{\rho}_{\mathbf{x}} \sim L^{-\alpha}) \propto L^{\hat{f}(\alpha) - \hat{d}} \;, \tag{S.2}$$

with $\hat{f}(\alpha)$ denoting the multifractal spectrum of the projected density. To test the validity of Eq. (S.2) and the size independence of the multifractal spectrum $\hat{f}(\alpha)$, we computed an ensemble of critical eigenstates and extracted $\hat{f}(\alpha)$ from it for system sizes in the range of $50 \leq L \leq 120$. The results summarized in Fig. S1 evidence the system-size independence of $\hat{f}(\alpha)$ for $\alpha \leq 2.4$, while the maximum being shifted from $\alpha = 2$ indicates the anomalous scaling of the typical projected density, $\hat{\rho}_{\rm typ} \sim L^{-\alpha_{\rm max}}$.

As shown in Fig. S1, both the $d = 3$ dimensional and the projected $\hat{d} = 2$- dimensional critical wave function densities display power-law correlations

$$C^{(1)}(\mathbf{r}) \propto |\mathbf{r}|^{-y_1} \;, \quad \text{and} \quad \hat{C}^{(1)}(\mathbf{x}) \propto |\mathbf{x}|^{-\hat{y}_1} \;, \tag{S.3}$$

with the critical exponents of the correlation functions are related as

$$\hat{y}_1 = y_1 - 1 \;. \tag{S.4}$$

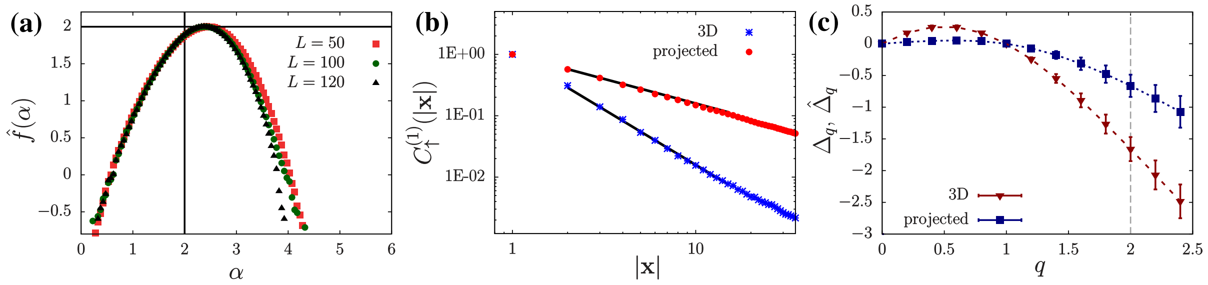

Supplementary Figure S1. Multifractal spectrum of the projected critical states of an ensemble of disordered samples with $W_c/J = 16.5$, and linear system sizes up to $L = 120$. **(a)** Multifractal spectra $\hat{f}(\alpha)$ calculated from the projected density for different system sizes. The high-amplitude (small α) part of the curves are found to be system-size independent. At the low-amplitude (large α) part finite size effects make the spectra slightly system-size dependent. **(b)** Density-density correlations for the three-dimensional density and the projected density of the critical state. Continuous black lines show power-law fits with exponents $y_1 = 1.82(1)$ and $\hat{y}_1 = 0.832(3)$. **(c)** Anomalous dimensions Δ_q and $\hat{\Delta}_q$ for the three-dimensional and the projected densities, respectively, extracted from the Legendre transforms of the corresponding $f(\alpha)$ and $\hat{f}(\alpha)$ functions. The relations $y_1 = -\Delta_2$ and $\hat{y}_1 = -\hat{\Delta}_2$ are satisfied within error bars.

To verify this connection we rewrite the correlation function of the projected densities as

$$\hat{C}^{(1)}(\mathbf{x}) = \overline{\hat{\rho}_{\mathbf{x}}\hat{\rho}_{\mathbf{0}}} = \int dz \int dz' \; \overline{\rho_{\mathbf{r}}\rho_{\mathbf{r}'}} = \int dz \int dz' \; C^{(1)}(\mathbf{r}-\mathbf{r}') \;, \tag{S.5}$$

where $\mathbf{r} = (x, y, z)$, $\mathbf{r}' = (0, 0, z')$, and disorder averaging is denoted by overline. Inserting the critical three-dimensional correlation function yields

$$\hat{C}^{(1)}(\mathbf{x}) = \int dz \int dz' \left(|\mathbf{x}|^2 + (z-z')^2\right)^{-y_1/2} \sim |\mathbf{x}|^{-y_1+1} \int dZ \left(1 + Z^2\right)^{-y_1/2} \sim |\mathbf{x}|^{-y_1+1} \;, \tag{S.6}$$

where in the last step we assumed $|\mathbf{x}| \ll L$ and used $y_1 \approx 1.8 > 1$ to carry out the integral. As shown in Supp. Fig. S1, our numerics are compatible with the simple analytical result, (S.4).

As mentioned in the main text, the correlation exponents y_q and $\hat{y}_q$ can also be determined from the multifractal spectra $f(\alpha)$ and $\hat{f}(\alpha)$ (see Eqs. (5) and (6) in the main text [2] and Ref. [3]). Taking the Legendre-transform of $f(\alpha)$ [$\hat{f}(\alpha)$] yields $\tau_q = 3(q-1) + \Delta_q$ [$\hat{\tau}_q = 2(q-1) + \hat{\Delta}_q$]. Panel (c) in Supp. Fig. S1 shows the resulting anomalous dimensions Δ_q and $\hat{\Delta}_q$ for the three-dimensional and the projected two-dimensional densities, respectively. According to Eq. (6) in the main text, the relations to the exponents $y_q = 2\tau_q - \tau_{2q} + d$ and $\hat{y}_q = 2\hat{\tau}_q - \hat{\tau}_{2q} + \hat{d}$ imply $y_1 = -\Delta_2$ and $\hat{y}_1 = -\hat{\Delta}_2$, respectively. Our numerical results in panel (c) of Supp. Fig. S1 indeed support these relations.

2 Surface and projected multifractality

As it is shown in the previous section, the projection of a critical eigenstate results in a two-dimensional multifractal, with universal density fluctuations and density-density correlations. However, projection is not the only way that leads to a two-dimensional multifractal from a three-dimensional critical state. Surface multifractality is an other characteristic property of the critical state, that has been investigated in the literature [4,5]. To observe the specific density fluctuations that lead to surface multifractality, one has to prescribe open boundary conditions in (S.1) in one or more directions. The density fluctuations of critical states close to the boundaries differ strongly from that of the bulk [5].

To compare the surface and projected multifractal spectra we study critical eigenstates of the Anderson model (S.1) where periodic boundary conditions are only prescribed only in the directions x and y while in the direction z we prescribe open boundary conditions. The probability density function of the density ρ_{surf} at the boundary is expected to scale as

$$P(\rho_{\text{surf}} \sim L^{-\alpha}) \propto L^{f_{\text{surf}}(\alpha)-d} \;. \tag{S.7}$$

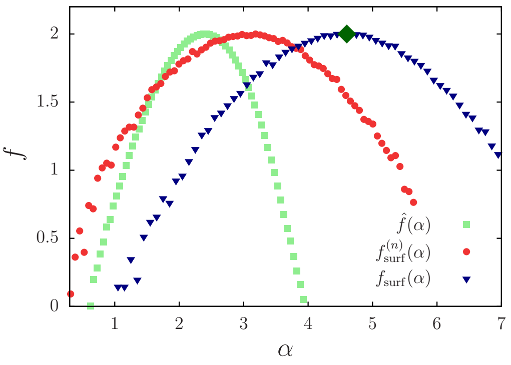

Supplementary Figure S2. The Normalized and not normalized surface multifractal spectra are shown by red circles and blue triangles respectively. The maximum of the spectrum $f_{\rm surf}(\alpha)$ is highlighted in order to emphasize that the position of this maximum is in good good agreement with the results of Ref. [5]. The projected multifractal spectrum $\hat{f}(\alpha)$ is shown by light green squares.

Using the method of Ref. [6], the surface multifractal spectrum $f_{\rm surf}(\alpha)$ can be extracted from the probability distribution of the densities at the boundary. It is important to note that in the case of three dimensional densities box-averaging is necessary to reveal universal multifractal properties [6], as it is also emphasized in the next section. Box sizes $l = 5a$ are used to extract the surface multifractal spectrum from critical eigenstates in disorder samples with linear system size $L = 100a$. The surface multifractal spectrum $f_{\rm surf}(\alpha)$ is shown in Supp. Fig. S2. The position of the maximum $\alpha^{\rm surf}_{\rm max} \approx 4.6$ of the surface multifractal spectrum $f_{\rm surf}(\alpha)$ is in good agreement with the finite-size scaling exponent of the typical value of the surface density [5].

The surface multifractal spectrum, as defined in Eq.(S.7), is not directly comparable with the projected multifractal spectrum $\hat{f}(\alpha)$, because the densities in the layer close to the boundary are not normalized to one, while the projected density is properly normalized. The “normalized” surface multifractal spectrum $f^{(n)}_{\rm surf}(\alpha)$, that is extracted after normalizing the densities at the layer close to the boundary to one, is also shown in Supp. Fig. S2. However, in spite of normalization, we find that the surface multifractal spectrum differs from the projected multifractal spectrum $\hat{f}(\alpha)$.

3 Three dimensional multifractality of the Gross-Pitaevskii wave function

Multifractal properties of the $d = 3$ dimensional Gross-Pitaevskii wave function have also been tested with findings similar to the ones presented in the main text [2]. The spectra in Supp. Fig. S3 were determined from the distributions of the Gross-Pitaevskii densities $\rho_{\mathbf{r},\uparrow}$. In the three-dimensional case box-averaging is necessary to reveal the universal multifractal spectrum [6]. For the Gross-Pitaevskii wavefunction we used $L = 40a$ and box sizes $l = 2a$, while for the critical eigenstates $L = 100a$ and $l = 5a$, yielding the same effective system size $L/l = 20$ in both cases. Similarly to the projected densities' multifractal spectrum, the high-amplitude (small α) part of the Gross-Pitaevskii multifractal spectrum remains very close to the one extracted from single critical eigenstates, in spite of the presence of weak interactions during time evolution. The measured multifractal spectrum distorts – similarly to the projected case – as we increase the interaction strength up to $U = 12J$ while keeping the atom density fixed. Deviations in the low-amplitude (large α) part may be explained by the fact that the Gross-Pitaevskii wave function is not a single eigenstate, but a superposition of several eigenstates of the self-consistent Hamiltonian. The $d = 3$ dimensional density-density correlations of the cloud can also be collapsed on the localized side of the transition by just rescaling the length and the wave function amplitudes (see Supp. Fig. S3 (b)).

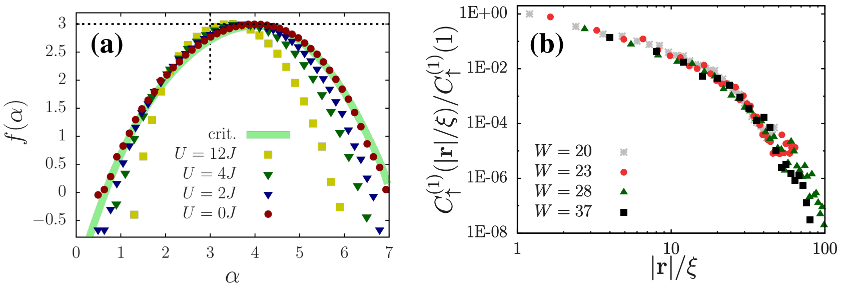

Supplementary Figure S3. **(a)** Multifractal spectrum extracted from the probability distribution of the three-dimensional density $\rho_{\mathbf{r}\uparrow}$. The spectrum measured after a pulse for non-interacing bosons (red circles) follows closely the spectrum obtained from the critical state (thick green line). Interactions mix in several eigenstates and deform the spectrum $f(\alpha)$ in the region of rare events (blue triangles). **(b)** Rescaled correlation function of the three-dimensional densities $\rho_{\mathbf{r}\uparrow}$ for different disorder strengths but fixed excitation frequency $\omega \approx 8J/\hbar$ and interaction strength $U = 2J$ in the localized regime.

4 Strong measurement of the projected atom density

As briefly discussed in the main text [2], direct measurement of the projected density $\hat{\rho}_{\mathbf{x}\uparrow}$ is difficult because most imaging techniques perform strong quantum measurements on the spatial positions of the individual atoms. Therefore atoms appear as dots in the images. In a given measurement the expectation value of the atoms that are found in the projected position $\mathbf{x}$ is simply $\hat{\rho}_{\mathbf{x}\uparrow}$. Since – assuming Poissonian statistics – the relative fluctuation of the measured particle number at site $\mathbf{x}$ from its average is simply $\hat{\rho}_{\mathbf{x}\uparrow}^{-1/2}$, measuring small densities accurately in single-shot experiments is impossible. One can overcome this difficulty by preparing the same condensate wave function multiple times. The expectation value of the total number of atoms at the projected position $\mathbf{x}$ is then $N_M \hat{\rho}_{\mathbf{x}\uparrow}$, with N_M denoting the total number of measurements. Thereby, relative fluctuations are reduced to $(N_M \hat{\rho}_{\mathbf{x}\uparrow})^{-1/2}$. Fortunately, since α is a logarithmic function of the density, the spectrum $\hat{f}(\alpha)$ can be reconstructed even from a rough estimate of $\hat{\rho}_{\mathbf{x}\uparrow}$ with only 10-20% relative error.

We emulated the statistical effects of strong quantum measurements on the Gross-Pitaevskii wave function by assuming an independent Poisson distribution of expectation value $\langle N_{\mathbf{x}} \rangle = \hat{\rho}_{\mathbf{x}\uparrow}$ for the measured number of atoms, $N_{\mathbf{x}}$ at each projected position $\mathbf{x}$. The measured single shot densities were then averaged, and the $\hat{f}(\alpha)$ multifractal spectrum was calculated from the averaged density. As shown in panel (a) of Supp. Fig. S4, performing roughly 1,000 single-shot measurements for identically prepared condensates is enough to reveal the desired $\hat{f}(\alpha)$ function for $\alpha \lesssim 3$, i.e. to see clearly the nontrivial position $\alpha_{\max}$ of the maximum of $\hat{f}(\alpha)$. However, already $N_M = 100$ single shot experiments are enough to measure the entire projected spectral function in the regime $\alpha \lesssim 2,4$.

To perform multiple measurements on the same state, one must be able to prepare the same condensate wave function multiple times. The nonlinearity induced by the $U_{\uparrow\downarrow}$ and $U_{\downarrow\downarrow}$ interaction terms makes the quantum state sensitive to the total number of atoms. Since fluctuations in the total atom number are unavoidable, we also tested the stability of the final multifractal spectrum against particle number fluctuations. As demonstrated in panel (b) of Supp. Fig. S4, even a 10% change in the initial density leaves the final state almost untouched.

5 Impact of interactions on the mobility edge

In our simulations we study the Anderson localization of the bosons in the hyperfine state $\uparrow$. These particles are subject both to the external random potential $V_\uparrow$ and the background potential $V_{\uparrow\downarrow} = U\,|\Psi_\downarrow|^2$ caused by the interaction with the $\downarrow$ particles. As we show in this section, the background potential of the $\downarrow$ particles is not uniform, therefore it may shift the position of the localization transition. However, we find that the magnitude of this potential is much smaller than the one of the external disorder, therefore the shift of the mobility edge is expected to be small.

In panel (a) of Supp. Fig. S5 a slice of the background potential $V_{\uparrow\downarrow}$ is shown close to the localization

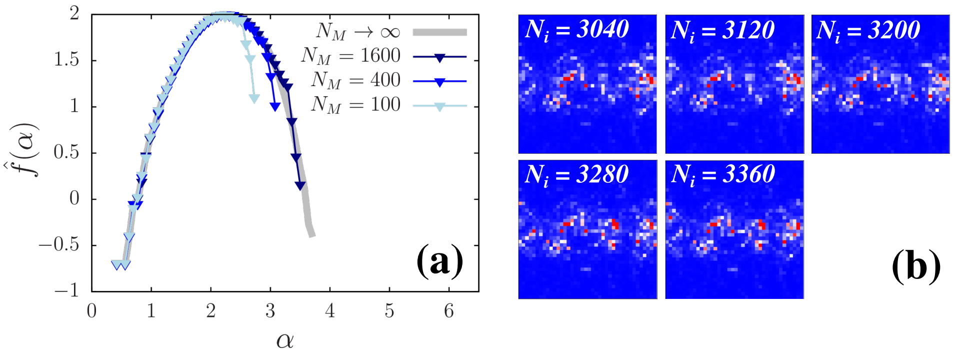

Supplementary Figure S4. **(a)** Reconstruction of the $\hat{f}(\alpha)$ multifractal spectrum from different number (N_M) of emulated strong measurements. While already $N_M \approx 100$ measurements reveal the small α part of the function, with $N_M \gtrsim 1000$ measurements almost the whole $\hat{f}(\alpha)$ becomes visible. **(b)** Projection images from the final condensate with different initial particle numbers N_i, while keeping the disorder potential, and RF frequency fixed. The final state and thus the multifractal spectrum remain almost unchanged even for particle number fluctuations of 10%.

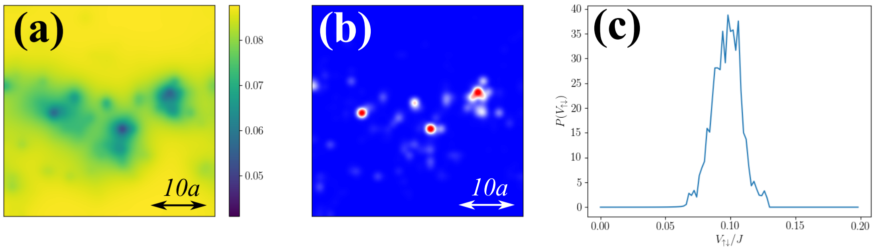

Supplementary Figure S5. **(a)** A slice of the background potential $V_{\uparrow\downarrow}$ is shown for excitation frequency $\hbar\omega = 7.18J$, disorder strength $W = 17J$ and interaction strength $U = 2J$. The colorbar shows the magnitude of the potential in units of J. **(b)** The corresponding slice of the density of the excited $\uparrow$ particles. The features of background potential in panel (a) resemble the excited $\uparrow$ states density profile. **(c)** The probability distribution of the background potential, averaged for the excitation frequencies ω.

transition, for a representative excitation frequency ω. Because of the repulsion between the $\uparrow$ and $\downarrow$ particles the background potential resemble the spatial structure of the excited $\uparrow$ state (see Supp. Fig. S5b). The magnitude of the spatial differences in background potential are as small as $\delta V_{\uparrow\downarrow} \approx 0.05J$ (see also Supp. Fig. S5c). In our simulations the critical regime is approached from the localized side of the transitions, and multifractality of slightly localized states at $W = 17J$ is studied, while the critical disorder strength around the band center is $W_c = 16.5J$ [6]. The smallest studied distance to the critical point is therefore $W - W_c = 0.5J$ that is an order of magnitude larger than the fluctuations of the background potential, and thus we do not observe any shift in the mobility edge. To observe this shift one should set the disorder strength closer to the critical value, while at the same time the spectral resolution should be much better to avoid spatial overlaps between the less localized eigenstates.

References

[1] Bollhöfer, M. & Notay, Y. JADAMILU: a software code for computing selected eigenvalues of large sparse symmetric matrices. *Computer Physics Communications* **177,** 951-964 (2007).

[2] Main text of this paper.

[3] Evers, F. & Mirlin, A. D. Anderson transitions. *Rev. Mod. Phys.* **80,** 1355-1417 (2008).

[4] Subramaniam, A. R., Gruzberg, I. A., Ludwig, A. W. W., Evers, F., Mildenberger, A., and Mirlin, A. D. Surface Criticality and Multifractality at Localization Transitions. *Phys. Rev. Lett.* **96,** 126802 (2006)

[5] Monthus, C. & Garel, T. Statistics of renormalized on-site energies and renormalized hoppings for Anderson localization in two and three dimensions. *Phys. Rev. B* **80,** 024203 (2009).

[6] Rodriguez, A., Vasquez, L. J., & Römer, R. A. Multifractal analysis with the probability density function at the three-dimensional Anderson transition. *Phys. Rev. Lett.* **102,** 106406 (2009).
